# Immunometabolic depression: from conceptualization towards implementation

**DOI:** 10.1192/j.eurpsy.2023.51

**Published:** 2023-07-19

**Authors:** B. Penninx, Immunometabolic depression: from conceptualization towards implementation

**Affiliations:** Amsterdam UMC, Amsterdam, Netherlands

## Abstract

**Abstract:**

The burden on society by depression is undisputable, partly due to a chronic course pattern and depression’s large heterogeneity that contributes to non-response to standard treatments. Using data from the Netherlands Study of Depression and Anxiety (NESDA, www.nesda.nl), Penninx will illustrate both points. When examining the course of depression, especially when also considering the transitions into other affective disorders over time, chronicity is clearly more the rule than the exception (Verduijn et al. BMC Med 2017). Considering depression’s heterogeneity could lead to precision psychiatry approaches that help reduce depression’s chronicity. Immuno-metabolic dysregulations seem to vary as a function of depression heterogeneity: dysregulations map more consistently to “atypical” neurovegetative symptoms reflecting altered energy intake/expenditure balance (hyperphagia, weight gain, hypersomnia, fatigue and leaden paralysis). Findings are confirmed when utilizing genome-wide gene expression as well as DNA information (Milaneschi et al. Biol Psychiatry 2020). Preliminary treatment studies suggest that the presence of immuno-metabolic dysregulations in depression moderates antidepressant effects of standard or novel (immunomodulatory) interventions. An immuno-metabolic depression dimension could dissect depression’s heterogeneity and potentially match depressed subgroups to treatments with higher likelihood of clinical success.

**Disclosure of Interest:**

None Declared

